# Single-cell profiling deciphering cholesterol metabolism dysregulation in metastatic uveal melanoma and implicating SLC45A2 in its prognosis

**DOI:** 10.3389/fimmu.2025.1660268

**Published:** 2025-10-01

**Authors:** Yiming Zhou, Yuan Cao, Zhaohui Li, Xinlan Lei, Qiao Gao, Wen Yao, Jian Guan, Guojing Lu, Haiyu Deng, Lanyue Zhang, Xiaoxi Deng, Zhen Chen, Yiqiao Xing

**Affiliations:** ^1^ Department of Ophthalmology, Renmin Hospital of Wuhan University, Wuhan, China; ^2^ Department of Ophthalmology, Aier Eye Hospital of Wuhan University, Wuhan, China; ^3^ Department of Ophthalmology, Northern Jiangsu People’s Hospital, Yangzhou University, Yangzhou, China; ^4^ Department of Ophthalmology, First Affiliated Hospital of Harbin Medical University, Harbin, China; ^5^ Joint Shantou International Eye Center of Shantou University and The Chinese University of Hong Kong, Shantou, China

**Keywords:** uveal melanoma, cholesterol metabolic process, single - cell sequencing, SLC45A2, cell-cell communication

## Abstract

**Introduction:**

Uveal melanoma (UM) is the most common primary intraocular malignancy in adults, characterized by high metastatic potential, primarily to the liver. UM exhibits unique molecular drivers, such as GNAQ/GNA11 mutations, and a distinct immune microenvironment. Despite these insights, the correlation and underlying mechanisms of lipid and cholesterol metabolism in UM development and progression have been scarcely investigated.

**Methods:**

Single-cell data analysis and cellular communication analysis were employed to investigate the effect of cholesterol metabolism in UM and its relationship with cell-cell communication and tumor immunology. Single-cell data of UM were acquired from the GEO repository and analyzed using R software. Key genes associated with cholesterol metabolism were validated *in vitro* using the human metastatic uveal melanoma cell line C918 and MuM2B.

**Results:**

Single-cell analysis revealed cellular heterogeneity in primary and metastatic UM samples. Metastatic UM tumor cells exhibited significantly lower cholesterol metabolism scores compared to primary tumor cells. Cell-cell communication analysis indicated that the Cho-high group demonstrated significantly higher levels of communication number and intensity. The APP-CD74 pathway was found to have relatively higher outgoing ligand-receptor communication in the Cho-high group. GSEA analysis found the mTORC1 signaling pathway and oxidative phosphorylation pathway is correlated to the cholesterol metabolism scores in melanoma cells in the UM dataset. Through correlation analysis and Cox regression, the gene SLC45A2 was identified as correlated with overall survival in both the TCGA-UVM dataset and the GSE84976. *In vitro* experiments showed that SLC45A2 depletion attenuated the metastatic and proliferative capacities of uveal melanoma cells.

**Conclusion:**

Overall, this study provides the first single-cell level investigation of cholesterol metabolism in UM and identifies SLC45A2 as a potential key gene affecting UM progression by regulating cholesterol metabolism. These findings offer new perspectives on the role of cholesterol metabolism in UM and lay a foundation for future clinical applications.

## 1 Introduction

Uveal melanoma (UM) is the most common primary intraocular malignancy in adults. Despite advances in clinical and basic research, UM is highly metastatic, and almost fifty percent of patients develop metastatic UM, which mostly occurs in the liver (1). The molecular characteristics and metastatic patterns of UM are significantly different from cutaneous melanoma. Although local treatments (such as proton radiotherapy) can effectively control the primary tumor, about 50% of patients still develop liver metastases, and the response rate to immune checkpoint inhibitors is extremely low (2). Unlike cutaneous melanoma, UM exhibits unique molecular driver mechanisms, such as GNAQ/GNA11 mutations, and a distinct immune microenvironment. Tumor-infiltrating lymphocytes are scarce, and M2-type tumor-associated macrophages predominate, creating a highly immunosuppressive microenvironment (3, 4). Despite all these insights, scant research has been done to investigate the correlation and underlying mechanisms of lipid and cholesterol metabolism in UM development and progression.

Abnormal cholesterol metabolism is an important feature of the tumor microenvironment (5–8). The relationship between melanoma pathogenesis and cholesterol metabolism has attracted increasing attention in recent years. Studies have shown that cholesterol and its metabolites play a significant role in the development and progression of melanoma. First, it has been pointed out that the cholesterol metabolite 27-hydroxycholesterol can promote melanoma growth by activating estrogen receptor α. This mechanism indicates a potential link between cholesterol metabolism and melanoma, especially under high-cholesterol diet conditions, where 27-HC may become an important factor in melanoma progression (9). Despite all these efforts, there is still a lack of studies indicating the potential significance of cholesterol metabolism in the development and progression of UM.

In this study, we combined single-cell data analysis with cellular communication analysis to investigate the effect of cholesterol metabolism in uveal melanoma and its correlation with cell-cell communication and tumor immunology. Further *in vitro* validation of the key gene associated with cholesterol metabolism in UM was conducted, highlighting the significance of cholesterol metabolism in the progression and prognosis of UM.

## 2 Materials and methods

### 2.1 Data acquisition and single-cell analysis

The single-cell data of Uveal Melanoma were acquired from the GEO repository (www.ncbi.nlm.nih.gov/geo) with the accession number GSE139829 and analyzed using R software (version 4.4.1) and the *Seurat* package (version 5.1.0). Single-cell data integration was conducted using the package *harmony* (version 1.2.1). The bulk sequencing of UM patient data, combined with their survival data, was downloaded from the TCGA database (https://portal.gdc.cancer.gov/) and the GEO database (accession number: GSE84976), respectively.

### 2.2 Single-cell data scoring

A total of five methods were used in scoring the single-cell data. The R packages used in this process were the package AUCell (version 1.14.0), *Ucell (Version 2.10)*, *irGSEA (Version 1.1.3)*, and *ssGSEA* (version 2.0). Data visualization was realized using the Package ggplot2 (version 3.5.1) and ggpubr (version 0.6.0). The information of the cholesterol metabolism-associated gene set was downloaded from the MSigDB database (https://www.gsea-msigdb.org/gsea/msigdb) and was listed in **Supplementary Table S1**.

### 2.3 Cell-cell communication and single-cell data differential analysis

The cell-cell communication analysis was conducted using the R package *CellChat* (version 1.6.1), and data visualization was conducted based on the same package. Function *FindMarkers* from the package *Seurat* was used to conduct differential analysis of single-cell data. The package ggplot2 (version 3.4.4) was used to draw the scatter plot for single-cell data.

### 2.4 GSEA analysis

Gene set enrichment analysis (GSEA) for pathways correlated to cholesterol metabolism scores was performed using R package *ClusterProfiler (version 4.14.0)* and *GseaVis(version 0.1.1).*

### 2.5 Cell culture, transfection, qPCR, and western blotting assays

Experimental validation was conducted *in vitro*. Briefly, Human metastatic uveal melanoma cell line C918 (CL-0264 Procell Inc., Wuhan, China) and MuM2B (FH-1158 Fuheng Biotechnology Inc., Shanghai, China) was cultured in RPMI-1640 medium supplemented with 10% fetal bovine serum. For siRNA-mediated gene knockdown experiments, C918 cells were seeded in 6-well plates at a density of 2.5*10^5^ cells per well. Cells were seeded and cultured for 24 hours to achieve 30%-50% confluency before transfection. siRNA targeting the gene of interest (si-SLC45A2; Tsingke Biotechnology) and non-targeting control siRNA (si-NC, Tsingke Biotechnology, Beijing, China) were complexed with the transfection reagent *Lipofectamine 2000™* (Invitrogen, Shanghai, China) according to the manufacturer’s protocol. The sequences of the siRNAs were listed in **Supplementary Table S2**. The methods of conducting qRT-PCR and Western Blotting have been fully described in our previous works (10, 11), and the forward and reverse primers for gene SLC45A2 were listed in **Supplementary Table S3**. Additionally, polyclonal antibodies for SLC45A2 western blotting were purchased from Proteintech (Proteintech, Wuhan, China, CatNo: 10453-1-AP).

### 2.6 Statistical analysis

For data following a normal distribution, the t-test and one-way ANOVA are used to compare between groups; for data following a non-normal distribution, the Wilcoxon test and Mann-Whitney U tests are conducted. P<0.05 is considered statistically significant.

## 3 Results

### 3.1 Single-cell analysis revealed cellular heterogeneity in primary and metastatic uveal melanoma samples

**Figure 1:**
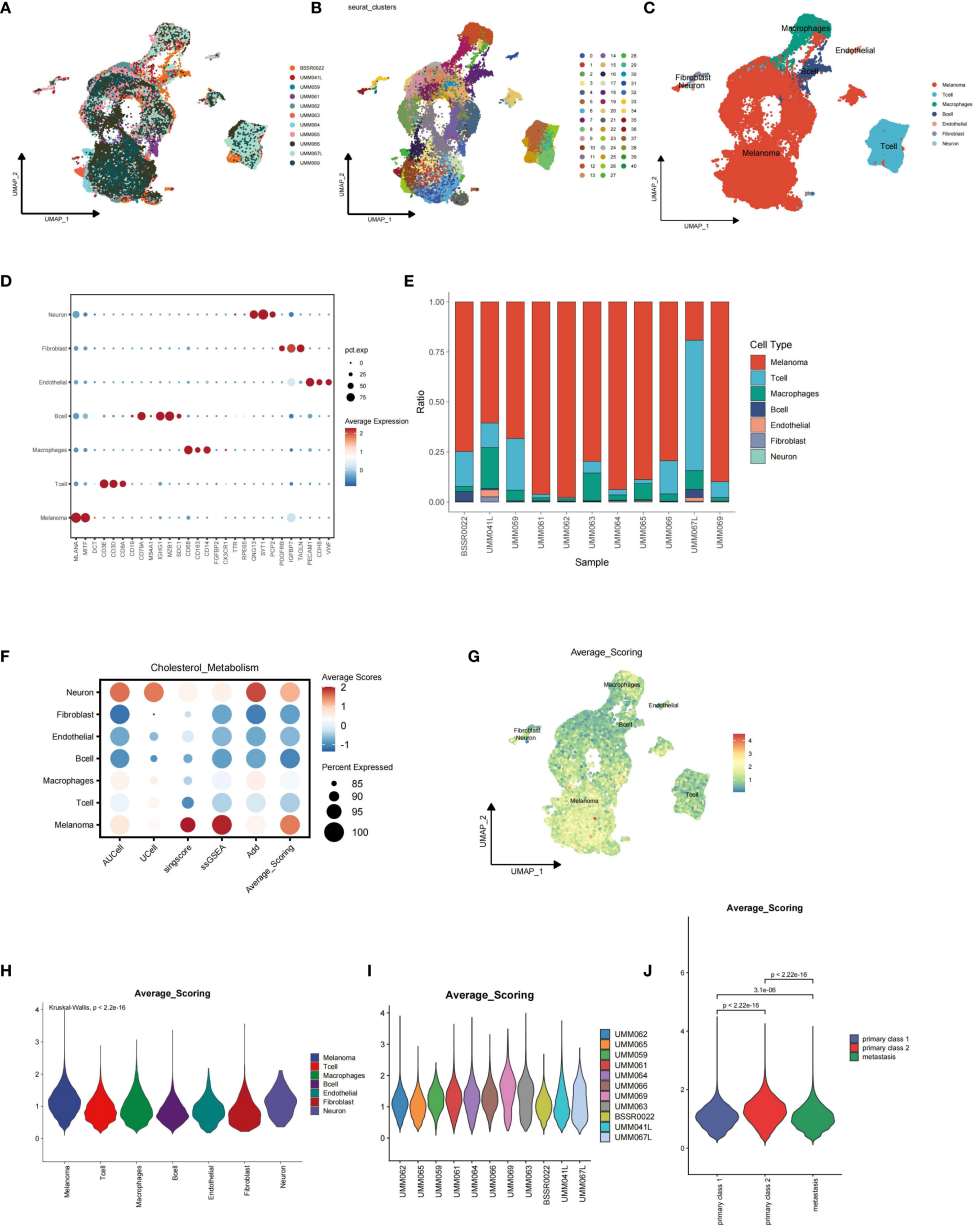
Integration of single-cell sequencing data from the GSE139829 dataset and single-cell data scoring for cholesterol metabolism using five different algorithms. **(A)** Batch effects between samples were eliminated using *harmony* algorithm. **(B-D)** Identification of distinct cell types, including melanoma cells, T cells, macrophages, B cells, endothelial cells, fibroblasts, and neural cells, based on their specific marker genes. **(E)** Proportion stack plot illustrating the relative abundance of different cell types across samples, highlighting a higher proportion of T cells in metastatic uveal melanoma samples (BSSR0022, UMM041L, and UMM067L) compared to primary samples (UMM062, UMM065, UMM059, UMM066, UMM061, UMM069, UMM064, and UMM063). **(F)** Dot-plot showing the Average scores across distinct cell types, with the color representing the scores and dot size showing the percentage cells with effective scoring. **(G)** Feature plot depicting cholesterol metabolism scores for each cell. **(H)** Violin plot showing the distribution of scores across major cell types. **(I, J)** Violin plots showing the distribution of cholesterol metabolism scores for melanoma cells across samples, with metastatic melanoma cells exhibiting lower scores.

Single-cell analysis combined with gene set scoring methods was applied to investigate the activity of cholesterol metabolism in uveal melanoma tissues. The dataset GSE139829 contained 11 uveal melanoma samples, including 9 primary samples and 2 metastatic samples. All data were merged and integrated to remove batch effect (**Figure 1A**). All cells were identified as melanoma cells, T cells, Macrophages, B cells, endothelial cells, fibroblasts, and neural cells based on their marker genes (**Figures 1B–D**). According to the phenotype data provided by the original dataset, samples UMM062, UMM065, UMM059, UMM066, UMM061, UMM069, UMM064, and UMM063 are primary uveal melanoma samples, while BSSR0022, UMM041L, and UMM067L are metastatic uveal melanoma samples. The proportion stack plot revealed a higher percentage of T cells in higher metastatic samples (**Figure 1E**).

### 3.2 Single-cell gene set scoring analysis revealed lower cholesterol metabolism scoring in metastatic uveal melanoma tumor cells

Single-cell gene set scoring analysis for cholesterol metabolism was conducted based on five distinct algorithms: AUCell, UCell, Singscore, ssGSEA, and AddModuleScore, and the average scoring for each cell and cell cluster was calculated accordingly. We found that melanoma cells ranked among the highest in cholesterol metabolism scoring of all cell types, indicating higher cholesterol metabolism activity in tumor cells than tumor-associated immune cells (**Figures 1F–H**). When we explored further into the melanoma cells of each sample, we found that the metastatic UM tumor cells showed significantly lower cholesterol metabolism scores than primary tumor cells (**Figures 1I–J**).

### 3.3 Cell-cell communication analysis revealed differences in cellular communications related to cholesterol metabolism

**Figure 2:**
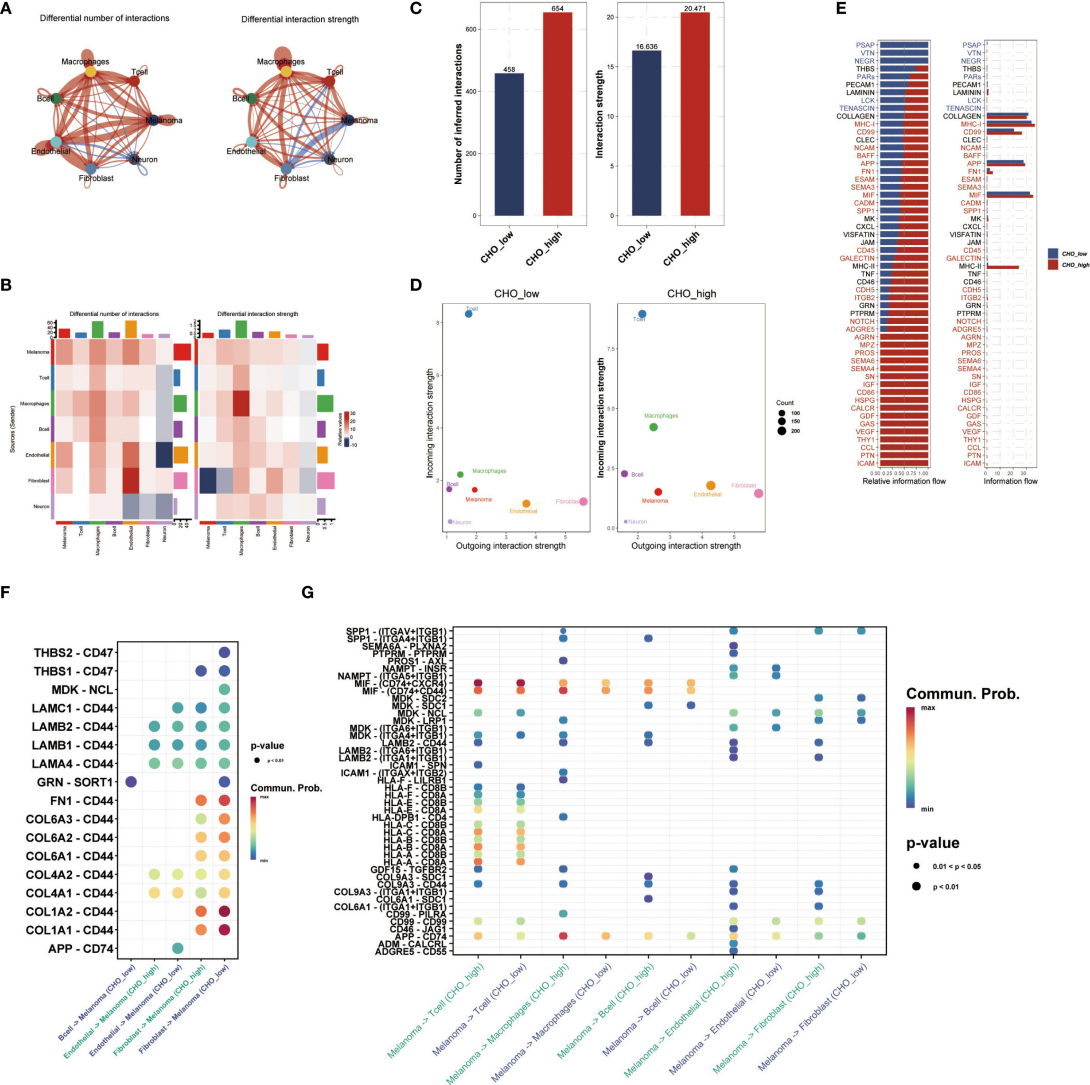
Cholesterol Metabolism is associated with Cell-Cell Communication in the UM Micro-environment. **(A)** Single-cell data were divided into Cho-high and Cho-low groups based on median cholesterol metabolism scores, followed by cell-cell communication analysis. **(B)** Analysis of communication strength based on specific cell types. **(C, D)** The Cho-high group showed higher communication numbers and intensity compared with the Cho-low group. **(E)** The bar plot showed the relative and actual information flow in single-cell data between the Cho-high and Cho-low groups. **(F, G)** Ligand-receptor interaction was quantified and visualized. In the Cho-high group, APP-CD74 outgoing communication was elevated, while the collagen-CD44 axis signal was higher in the Cho-low group.

Based on the median cholesterol metabolism score, all single-cell data were split into the high metabolism score group (Cho-high group) and the low metabolism score group (Cho-low group), and both groups were subjected to cell-cell communication analysis. Differential analysis showed a higher amount of interactions between fibroblasts and endothelial cells in the Cho-high group (**Figure 2A**), and the interaction strength within macrophages in the Cho-high group also rose considerably (**Figure 2B**). Further explorations revealed that in general, the CHO-high group exhibited significantly higher levels of communication number and intensity (**Figures 2C–E**). We also found that in the higher metabolism score group, the outcoming ligand-receptor communication of APP-CD74 was relatively higher in the CHO-high group (**Figure 2F**), whereas in the CHO-low group the incoming chollagen-CD44 axis signal was generally higher than the CHO-high group (**Figure 2G**), indicating the correlation between Cholesterol metabolism with tumor microenvironment and immunosuppression.

### 3.4 Identification of cholesterol-metabolism-associated genes and pathways in uveal melanoma

**Figure 3:**
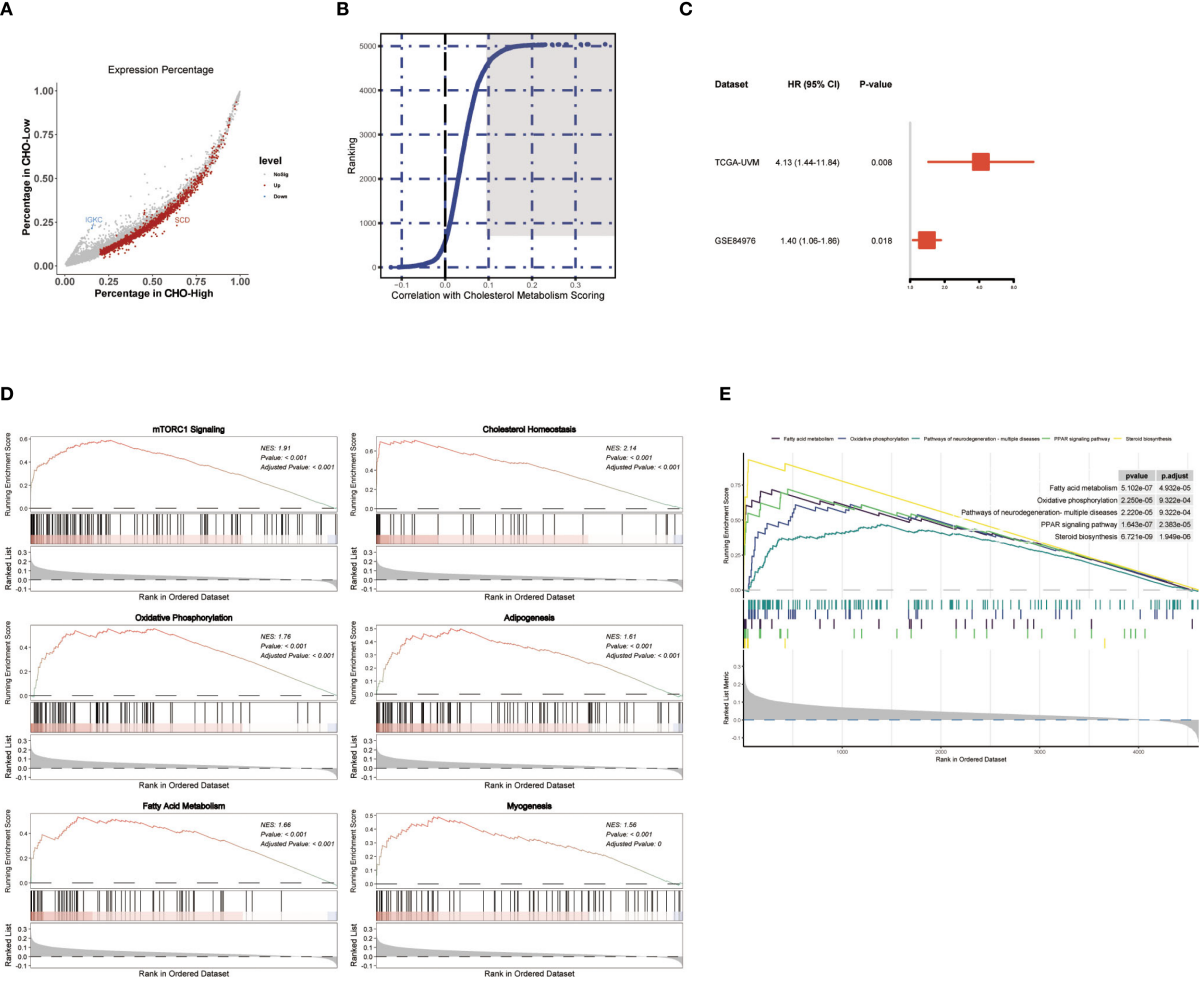
Identification of Cholesterol Metabolism Related Genes SLC45A2 Correlated to UM Prognosis. **(A)** Scatter plot showing genes with logFC>2 and P value<10–^5^ and expressed in more than 20 percent of melanoma cells in either of the two categories. **(B)** Correlation analysis for genes with average cholesterol metabolism scores in melanoma cells single-cell dataset GSE139829. The top 500 genes were marked in dark grey (**Figure 4B**). **(C)** Forest plot showing the Cox regression results of SLC45A2. SLC45A2 was correlated to overall survival in both the TCGA-UVM dataset and the GSE84976. **(D, E)** GSEA analysis was conducted after correlation analysis for all genes with average cholesterol metabolism scores in melanoma cells.

To further explore the potential molecular impact of cholesterol metabolism on affecting disease outcome, we screened for the genes that are both associated with UM prognosis and cholesterol metabolism scores. Firstly, all melanoma cells we separated into two categories: the high metabolism score group and the low metabolism score group based on the median cholesterol metabolism scores. Differential analysis of the two groups was conducted. Genes with log_2_FC>2 and P value<10–^5^ and expressed in more than 20 percent of melanoma cells in either of the two categories were selected (**Figure 3A**). Besides, a correlation analysis with average cholesterol metabolism scores was conducted, with the top 500 genes selected (**Figure 3B**). Consequently, genes that met both of the two aforementioned criteria were subject to Cox regression, and we found that gene SLC45A2 was correlated to overall survival in both the TCGA-UVM dataset and the GSE84976 (**Figure 3C**).

Based on the correlation analysis conducted in **Figure 3B**, we analyzed the pathways that were correlated to the cholesterol metabolism scores in the UM sample. GSEA analysis using MsigDB hallmark gene sets (**Figure 3D**) and Kyoto Encyclopedia of Genes and Genomes (KEGG) gene sets (**Figure 3E**) revealed that the “oxidative phosphorylation pathway” and the “mTORC1 signaling pathway” were strongly correlated to the cholesterol metabolism scores in melanoma cells in the UM dataset GSE139829.

### 3.5 SLC45A2 depletion attenuates metastatic and proliferative capacities in uveal melanoma cells

**Figure 4:**
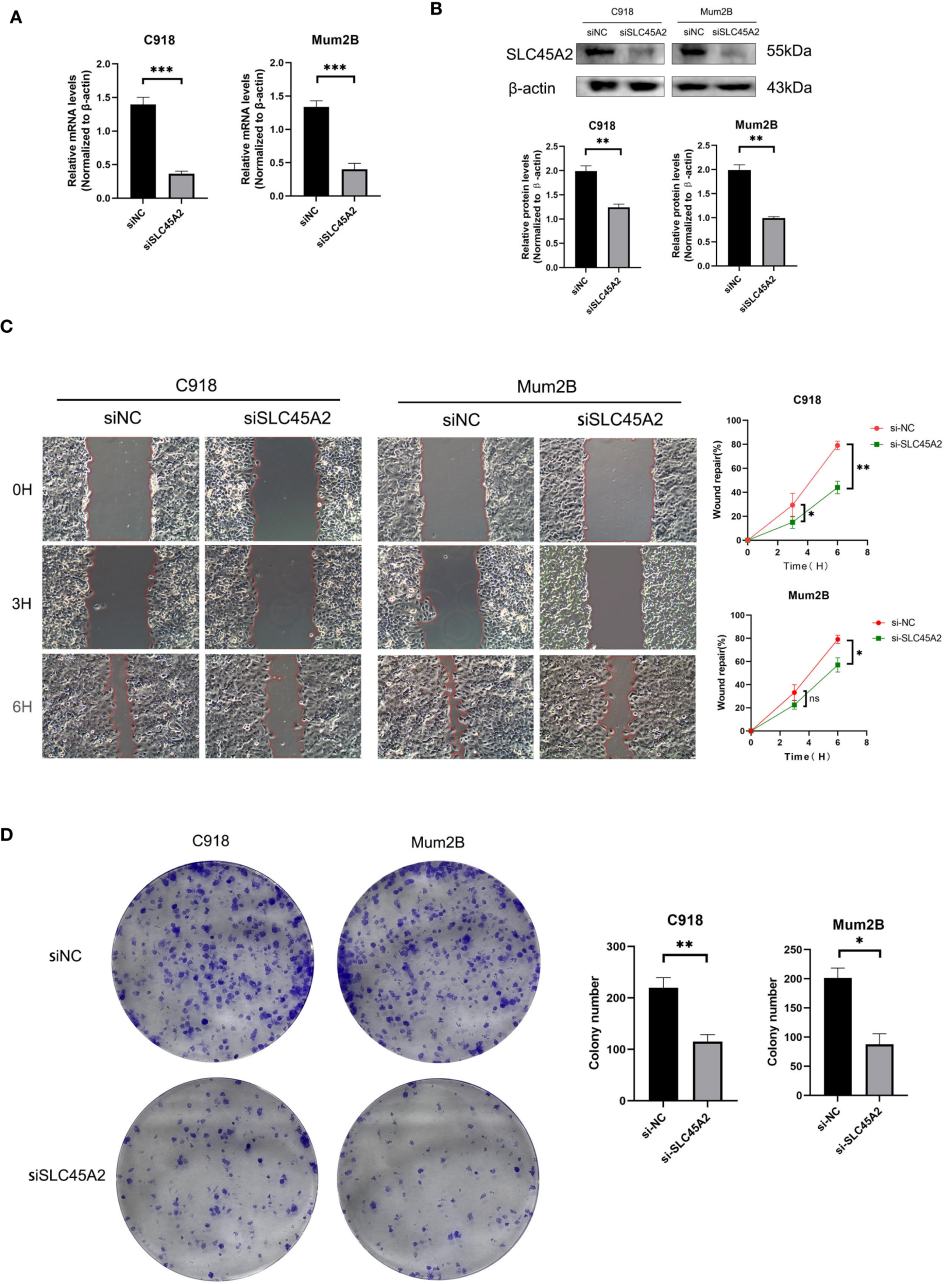
SLC45A2 knockdown inhibits cell migration and clonogenicity in uveal melanoma C918 and MuM2B cells. **(A, B)** qRT-PCR and Western blotting to evaluate the level of SLC45A2 mRNA and protein levels 48 h after transfection. All siRNA sequences could result in significant decrease in SLC45A2 mRNA and protein levels expression (P<0.001). **(C)** Transwell assay. The migration and invasion capacity of C918 and MuM2B cells decreased significantly after SLC45A2 knockdown. **(D)** Scratch-wound healing assay. A significantly slower wound healing rate was observed in cells with a decreased expression of SLC45A2 gene. All data were presented as the means ± SD of three independent experiments. (*P < 0.05, **P < 0.01, ***P < 0.001).

To verify the effect of SLC45A2 on the function of human uveal melanoma cell line C918 and MuM2B, we first validated the knockdown efficiency of SLC45A2 in the C918 and MuM2B cell lines by analyzing the expression level of SLC45A2 mRNA via qRT-PCR (**Figure 4A**) and the representative expression of SLC44A2 protein via Western blot (**Figure 4B**). The cells were transfected with siRNA targeting SLC45A2 or a non-targeting control siRNA (siNC). Compared with the siNC group, the mRNA and protein levels in the siRNA treatment groups were significantly reduced (**Figure 4A, B**, p < 0.05). The wound healing assay indicated impaired cell migration. Differential interference contrast microscopy images at 0 h and 3 h, and 6 h after scratching. Compared with the control cells (siNC group), the wound closure ability of the cells with SLC45A2 knockdown (siSLC45A2 group) was significantly reduced, indicating that their migration ability was inhibited (**Figure 4C**). The clone formation survival rate was evaluated by crystal violet staining. Compared with siNC, knockdown of SLC45A2 significantly reduced the number and size of cell colonies, confirming that SLC45A2 plays a key role in maintaining the proliferation potential (**Figure 4D**).

## 4 Discussion

Uveal melanoma is a malignant tumor with high metastatic potential. When studying uveal melanoma and its related molecular mechanisms, the regulation of metabolic pathways should be taken into account due to its vital role (12). Despite the findings that have validated the correlation between cutaneous skin melanoma and cholesterol metabolism (13), there is still a lack of research depicting the cholesterol metabolic profiles in uveal melanoma (14).

In this study, by harnessing the single-cell sequencing data, we constructed a cholesterol metabolic profile using five different scoring algorithms. Because the dataset contained both primary and metastatic UM samples, we found that the metabolic scores decreased significantly in the metastatic UM cells, implying the potential role that the metabolic pathway plays in UM metastasis. Notably, our study pioneered in cholesterol metabolism in UM despite a previous study indicating a correlation of lipid metabolism and cholesterol metabolism with cutaneous malignant melanoma (9, 15). Further studies based on cell-cell communication were carried out to investigate the correlation between cholesterol metabolism and intracellular communication. We discovered that certain cell-cell communication pathways were significantly higher in the higher metabolism group. Also, certain communication pathways between melanoma cells and T cells/Macrophages, like APP-CD74 pathways, were higher in the higher cholesterol metabolism group than in the lower cholesterol metabolism group, indicating the possible link between cellular communication pathways and cholesterol metabolism in UM.

GSEA analysis conducted in single-cell data identified that certain pathways, including the mTORC1 pathway, were associated with cholesterol metabolism in uveal melanoma. mTORC1 has been widely acknowledged to activate sterol regulatory element-binding proteins (SREBPs), thereby regulating cholesterol synthesis (16, 17). Targeting mTORC1 has been verified to inhibit melanoma cell growth and invasion (16). Therefore, our studies further provided bioinformatic evidence for the understanding of the mechanisms behind the cholesterol metabolism in UM pathogenesis.

Solute Carrier Family 45 Member A2 (SLC45A2), as a membrane-associated transporter protein, has attracted increasing attention in melanoma in recent years. Mutations in the SLC45A2 gene are associated with oculocutaneous albinism type 4 in various species and are also related to melanoma susceptibility (17, 18). SLC45A2 has high tumor selectivity and low potential for autoimmune toxicity. Studies have found that cytotoxic T lymphocytes (CTLs) targeting SLC45A2 can effectively eliminate most HLA-matched melanoma cell lines while significantly reducing the recognition of normal melanocytes (19). Moreover, certain single-nucleotide polymorphisms (SNPs) of the SLC45A2 gene are associated with melanoma susceptibility (20). In Southern European populations, the p.Phe374Leu variant of SLC45A2 is considered a strong protective factor for melanoma, especially in individuals without clinical risk factors (21). Similarly, in the French population, variations in SLC45A2 have also been found to have a protective effect against melanoma, and together with variations in the MC1R gene, they influence the risk of melanoma (22).

Studies have shown that the loss of function of the SLC45A2 gene is associated with melanoma susceptibility. As a proton/glucose exporter, the loss of SLC45A2 leads to acidification of the melanosome’s internal environment, thereby affecting the activity of tyrosinase, a key enzyme in melanin synthesis. Research has found that the loss of SLC45A2 significantly upregulates the activity of glycolytic enzymes and the PI3K/Akt signaling pathway, promoting glycolysis-dependent survival and metastasis of melanoma cells. These findings reveal the important role of SLC45A2 in regulating glucose metabolism in melanosomes and provide a new perspective on the metabolic regulation of melanoma (23). Therefore, the SLC45A2 gene plays a significant role in the occurrence and development of melanoma, and its variations not only affect melanin synthesis and metabolism but may also influence tumor progression by regulating intracellular metabolic pathways. Our studies provide new perspectives on the mechanism of SLC45A2 in melanoma and lay the foundation for future clinical applications.

In summary, this research was the first to investigate cholesterol metabolism in UM on the single-cell scale. We have also discovered the key gene SLC45A2, which may affect the progression of uveal melanoma by regulating related metabolisms such as cholesterol metabolism and glucose metabolism. Further researches are needed to explore the specific mechanism of cholesterol metabolism and its potential application in melanoma treatment. These studies will provide an important theoretical basis for the development of new treatment strategies.

## Data Availability

The raw data supporting the conclusions of this article will be made available by the authors, without undue reservation.
